# Genesis of solid bitumen

**DOI:** 10.1038/s41598-020-72692-2

**Published:** 2020-09-24

**Authors:** Hamed Sanei

**Affiliations:** Lithospheric Organic Carbon (LOC) Group, Department of Geoscience, Aarhus University, Høegh-Guldbergs gade 2, 8000C Aarhus, Denmark

**Keywords:** Geochemistry, Geology, Sedimentology

## Abstract

This paper presents a new schematic model for generation and timing of multiple phases of solid bitumen throughout the continuum of organic matter maturation in source and tight reservoir rocks. Five distinct stages in the evolution of solid bitumen are proposed: (1) *diagenetic solid bitumen* (or *degraded bituminite*), which is not a secondary maceral resulting from the thermal cracking of kerogen. Instead it is derived from degradation of *bituminite* in the diagenesis stage (Ro < 0.5%); (2) *initial-oil solid bitumen*, is a consolidated form of early catagenetically generated bitumen at the incipient oil window (Ro ~ 0.5–0.7%); (3) *primary-oil solid bitumen* is derived from thermally generated bitumen and crude oil in the primary oil window (Ro ~ 0.7–1.0%); (4) *late-oil solid bitumen* (solid-wax) is derived from the waxy bitumen separated from the mature paraffinic heavy oil in the primary- and late-oil windows; and (5) *pyrobitumen*, which is mainly a non-generative solid bitumen, is evolved from thermal cracking of the remaining hydrocarbon residue and other types of solid bitumen in the dry gas window and higher temperature (Ro > 1.4%). This model shows concurrence of multi-populations solid bitumen with oil, bitumen, and other phases of fluid hydrocarbon residue during most of the maturity continuum.

## Introduction

The evolution of organic matter immediately after sinking through the water column and deposition in sedimentary basins involves a series of post-burial bacterial activities and low temperature chemical reactions during the diagenesis stage, followed by thermal maturation and thermal cracking during the catagenesis and metagenesis phases. The low temperature reactions involving recently deposited organic matter lead to the formation of kerogen at the end of the diagenesis stage. The subsequent thermal cracking of kerogen during catagenesis and metagenesis lead to the formation of oil and gas, respectively^1–3^.

While the thermogenic generation of oil and gas is generally well understood, significant confusion remains over the description, timing, and genetic classifications of the intermediate and secondary hydrocarbon products. Bitumen and solid bitumen are secondary hydrocarbon products that are found in most source and tight reservoir rocks. They are formed during different stages of the post-burial organic matter evolution continuum. The highly confounded aspects of bitumen and solid bitumen are related to their genetic classification and the timing of their occurrences relative to onset of oil generation. Pre-oil and post-oil bitumens were long recognized by Taff^4^, Brooks^5^, and Silverman^6^. However, the seminal work of Curiale^7^ has prominently coined the genetic classification of solid bitumen into (1) pre-oil solid bitumen as “early-generation products of rich source rocks, probably extruded from their sources as a very viscous fluid, and migrated the minimum distance necessary to reach fractures and voids in the rock,” and (2) post-oil solid bitumen, which is the product of “the alteration of a once-liquid crude oil, generated and *migrated* from a conventional oil source rock, and subsequently degraded”. (see also Mastalerz et al.^8^ and references there in) (Table 1).

**Table 1:** Comparison between the current and new genetic classification of solid bitumen.

Maturity Stages^a^	Diagenesis Ro < 0.5%		Catagenesis 0.5% < Ro < 2.0%		
Hydrocarbon generation windows^b^	Pre-oil generation (immature) Ro < 0.5%	Incipient-oil generation (early oil) Ro ~ 0.5–0.7%	Primary-oil generation (peak oil) Ro ~ 0.7–1.0%	Post-oil generation (late oil /wetgas) Ro ~ 1.0–1.4%	Dry gas generation (overmature) Ro > 1.4%
Current classification^c^	Pre-oil Solid Bitumen		Post-oil Solid Bitumen		
**New classification**^**d**^					
* - Precursor bitumen:*	*Bituminite/ Amorphinite*	Bitumen (Exsudatinite)	Asphalt	Waxes	Hydrocarbon residue
* - Solid Bitumen:*	*Diagenetic Solid bitumen*^*e*^	*Initial-oil Solid Bitumen*	*Primary-oil Solid Bitumen*	*Late-oil Solid Bitumen*^*f*^	*Pyrobitumen*

^a^Organic maturity stages and their vitrinite reflectance ranges (Ro); ^b^hydrocarbon generation windows and their respective Ro thresholds^1,3,12,15,17^; ^c^existing genetic classification of solid bitumen based on Curiale^7,8^; ^d^new proposed genetic classification of bitumen and solid bitumen. ^e^degraded bituminite; ^f^solid-wax or ozocerite.

This classification has ambiguously used “pre-oil solid bitumen” for both diagenetically formed solid bitumen in immature source rocks as well as the catagenetically generated solid bitumen at the beginning of the oil window (but just before onset of crude oil)^7,8^ (Table 1). Although both types of reported solid bitumens are formed before generation of liquid crude oil, there is, however, a drastic difference in the nature, genesis, and timing of these two types of “pre-oil” solid bitumens. This ambiguity has creeped in the subsequent literatures resulting in indiscriminate use of “pre-oil” term for both diagenetic and early oil window solid bitumens^8–11^.

While Curiale’s^7^ “pre-oil” solid bitumen is mainly intended for the early thermal generation product of kerogen, this process takes place within the oil window at the “incipient oil generation” stage, defined by another seminal work^12^ (Table 1). Therefore, Curiale’s^7^ genetic term of “pre-oil” doesn’t conform to the correct hydrocarbon generation window. Lewan^12^ has used the “pre-oil” generation for a completely different thermal maturity stage than Curiale’s^7^ intended “pre-oil”. Lewan’s^12^ “pre-oil” window refers to the diagenetic stage of organic matter evolution, before the commencement of the oil generation window (Table 1). This simple contradiction in the application of “pre-oil” between the two influential studies has caused a great deal of confusion and consequently “pre-oil” bitumen/solid bitumen has been frequently used for two completely different products^7–9,11^.

Moreover, Curiale^7^ has classified “post-oil” solid bitumen as a single phase formed after the generation of crude oil (Table 1). The genetic classification of solid bitumen based on Curiale^7^ does not distinctly address the multiples phases and timing of the post-oil solid bitumen generations during the thermal maturation process.

Because solid bitumen is observed in source rocks across a wide range of thermal maturity, the use of the genetic terms pre-oil and post-oil has little useful meaning, and thus should be abandoned. The purpose of this paper is to propose an alternative scheme to classify the generation of solid bitumen. This paper proposes the first comprehensive schematic model showing the evolution of organic matter, and its secondary by-products, bitumen and solid bitumen, in relation to the oil and gas generations during thermal maturity. This model provides an explanation for the geochemical and petrographical occurrences of bitumen and solid bitumen through the continuum of diagenesis and catagenesis stages in organic-rich source and tight reservoir rocks.

## Functional definitions of bitumen and solid bitumen

### Bitumen

Bitumen is a highly viscous, semi-fluid to semi-solid hydrocarbon residue (FHR), which can be extracted by organic solvents (e.g., carbon disulfide)^3,13^. In organic petrology, bitumen is commonly observed in a dark brown color under white-reflected-light microscopy^13–15^. Bitumen is not fully consolidated and hence does not produce a polishable surface during the mechanical preparation of the rocks’ polished blocks^13,14,16^. Therefore, bitumen mostly absorbs the white light, which results in its dark brown appearance^13^. At a microscopic scale, bitumen is often disseminated parallel to the bedding fabrics of a rock or may sometimes be distributed as a fluid-like, intergranular, surface coating matter^13,15,17–19^.

Bitumen occurs as a secondary organic matter generated during catagenesis due to (1) thermal cracking of kerogen in the initial/incipient oil window (exsudatinite is likely to be the maceral equivalent of bitumen, which is formed from early thermal cracking of liptinitic kerogen)^12,15^ or (2) natural deasphalting and wax crystallization of liquid oil throughout the primary oil and late oil windows^1,3,20–22^. In thermally immature samples, where no thermal generation of bitumen has yet occurred, amorphous organic matter with similar petrographic characteristics as bitumen can be found. These bitumen-like organic matter are regarded as *bituminite* (or *prebitumen* or *amorphinite*). *Bituminite* is not a secondary maceral resulting from the thermal cracking of kerogen and instead it is derived from the primary deposition of flocculent protohydrocarbons or lipid organic matter^23–26^.

### Solid bitumen

Solid bitumen is a (semi)solid, opaque, amorphous, organic mass, derived from consolidation of bitumen (mineral bitumen^3^). Solid bitumen can be solid at the reservoir temperature or solidified because of drop in temperature below the pour point, once the sample is brought to surface^3^. Solid bitumen is primarily formed due to direct flocculation and precipitation of solid hydrocarbons from solution with the change in pressure–volume–temperature (PVT), natural deasphalting of crude oil, biodegradation, chemical dehydrogenation (oxidation), and devolatilization (thermal alteration) of bitumen and hydrocarbon residuum^3,22,27–36^.

While bitumen is well known within the context of organic geochemistry, solid bitumen is more specifically recognized in organic petrology. Solid bitumen is petrographically identified as semi-solid to solid amorphous organic matter. Due to its solid physical state, solid bitumen produces a polished surface, during the mechanical preparation of the rocks’ polished blocks. The polished surface of solid bitumen is capable of reflecting light and is seen as various shades of grey under white-reflected light microscopy^8,11,37^. The reflectivity of the surface of solid bitumen is a direct function of thermal maturity and is widely used as a thermal maturity proxy (solid bitumen reflectance: BRo%)^20,21,38,39^.

## New genetic model for generation of bitumen and solid bitumen

**Figure 1:**
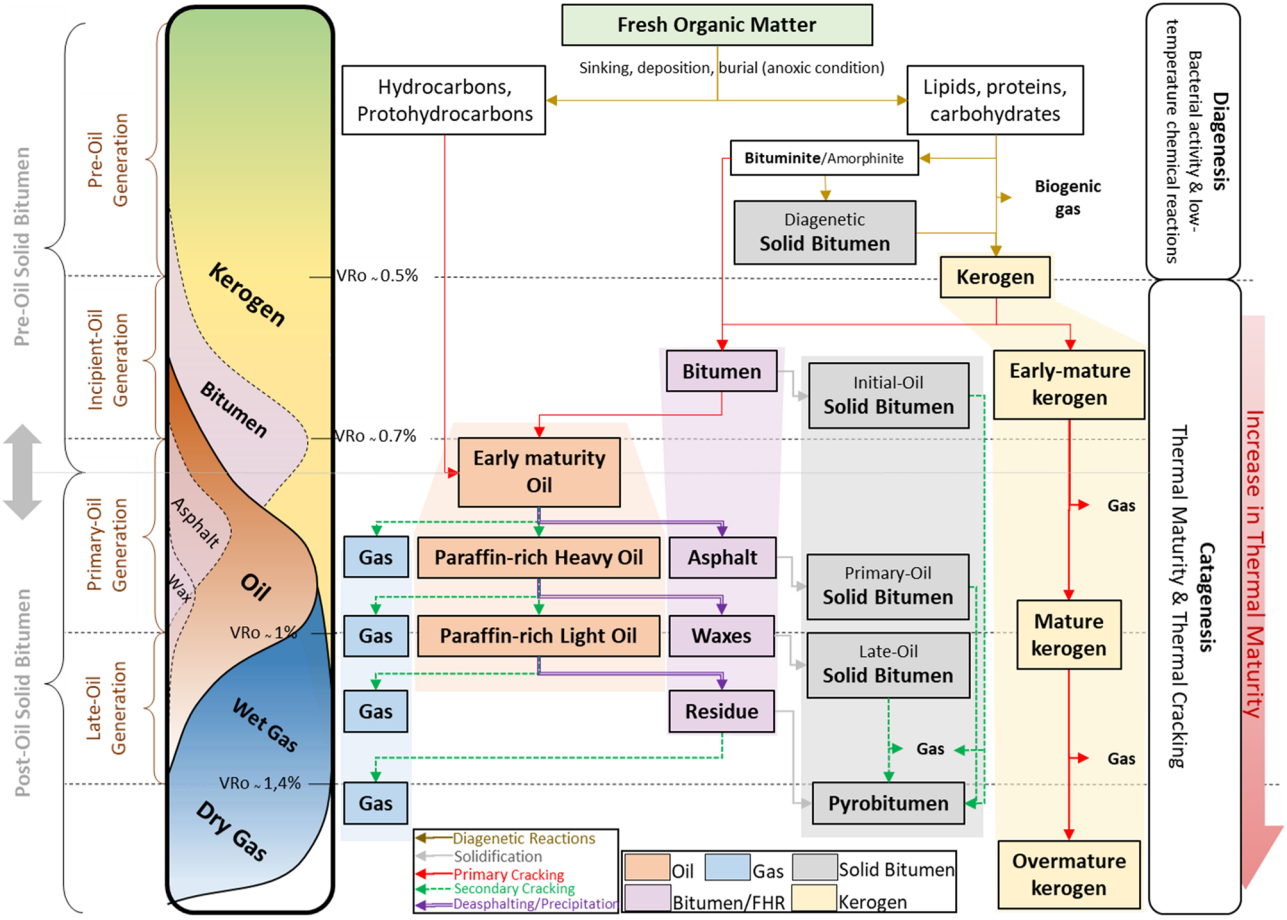
Genesis and timing of bitumen and solid bitumen throughout the continuum process of thermal maturity.

This paper presents a new classification and schematic model for the generation and timing of bitumen and solid bitumen during organic carbon thermal maturation in source/tight reservoir rocks (Table 1; Fig. 1). The post-burial maturation of organic matter follows two pathways: (1) an accumulation of the free proto-hydrocarbons from the buried organisms in the recent unconsolidated sediments, which can be readily converted to hydrocarbons. (2) Conversion of the lipids, proteins, and carbohydrates in the buried organisms into the kerogen at the end of the diagenesis stage (Ro ~ 0.5%)^3^ (Fig. 1).

During the catagenesis stage, the oil-generative portion of the kerogen undergoes consecutive thermal crackings that lead to formation of the entirely new hydrocarbon matters such as oil, gas, bitumen, and solid bitumen^1–3,20,21^. Lewan^12^ has divided thermal maturation of petroleum into four stages: (1) pre-oil generation (immature zone; Ro < 0.5%), (2) incipient-oil generation (Ro ~ 0.5–0.7%), (3) primary oil generation (Ro ~ 0.7–1.0%), (4) post-oil (late-oil) generation (Ro ~ 1.0–1.4%). Onset of crude oil (petroleum) is towards the end of incipient-oil window (Table 1; Fig. 1).

The gas- and non-generative portions of kerogen also undergo thermal alterations, which lead to structural deformations, increase in reflectivity, increase in porosity, and loss of generative potential. This non-generative, overmature kerogen, at the end of the catagenesis stage, is also regarded as inertinite or dead organic carbon^37,40^ (Fig. 1).

This study proposes a new genetic classification of solid bitumen, which includes the following five distinct types (Table 1; Fig. 1).

### Diagenetic solid bitumen (degraded bituminite)

*Diagenetic solid bitumen* (*degraded bituminite*) has similar optical properties under reflected-light microscope as solid bitumen. It is however not a secondary maceral resulting from the thermal cracking of kerogen and hence is not, by definition, considered a solid bitumen. This type of “pseudo” solid bitumen is frequently reported in immature (Ro < 0.5%) carbonaceous shales and fine siltstones^9–11,30,31^ and is derived from biodegradation and low-temperature alteration of *bituminite/amorphinite* in the diagenesis stage (Ro < 0.5%; in Lewan’s^12^ “pre-oil generation” window) (Table 1; Fig. 1).

Primary deposition of flocculent lipid components under oxygen-deficient bottom water conditions results in accumulation of an early diagenetic bituminous matter, regarded as *bituminite* in immature carbonaceous rocks^23–26,41,42^. Further biodegradation and other low temperature alteration of *bituminite* during the diagenesis stage can result in formation of *degraded bituminite* designated in this study as *diagenetic solid bitumen* (Table 1; Fig. 1)*.*

**Figure 2:**
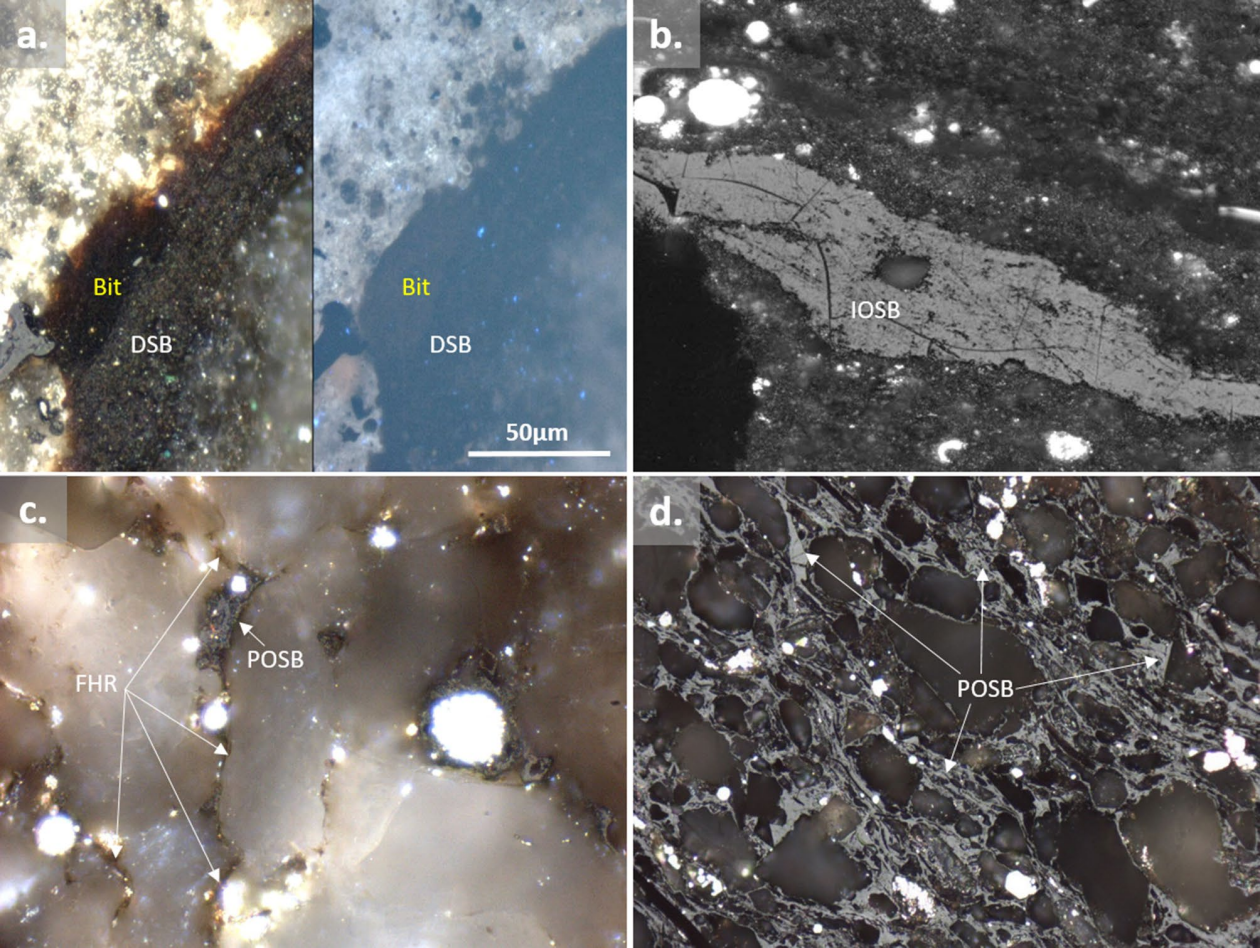
Incident-light, immersion oil, photomicrographs showing (**a**) left (white light), right (fluorescence light), *diagenetic solid bitumen* (DSB) (amorphous, granular, dark grey), which is developed from consolidation and degradation of *bituminite* (Bit; dark brown color) in an immature, Lower Cretaceous, calcareous mudrock of the Danish North Sea. (**b**) *Initial-oil solid-bitumen* (IOSB), which is characteristically associated with its precursor oil prone organic matter (alginate?); solid bitumen has occupied the place of its kerogen precursor (alginate?) with no evidence of flow and lacks of a pervasive migrations. Sediment matrix wraps around the IOSB indicating that the precursor organic matter deposited before lithification; (**c**) Coexistence of *primary-oil solid bitumen* (POSB) occupying the larger intergranular pore spaces, with the semi(fluid) hydrocarbon residue (FHR; brownish color) distributed in the interstices of the silt grains (Lower Triassic, Canadian Montney siltstone); (**d**) Pervasive migratory distribution of *primary-oil solid bitumen* (POSB) in the Lower Triassic, Canadian Montney tight reservoir.

*Diagenetic solid bitumen* is an opaque (or partly translucent), (semi)solid, amorphous organic mass, with a soft polishable surface, which is optically observed in dark grey color (in white reflected light microscopy) and resemble a weak to no fluorescence properties (in ultra violet reflected light microscopy)^11,30,31,41^ (Fig. 2a). *Diagenetic solid bitumen* is not expected to be from a migratory source but rather appears malleably deformed by compaction. Sediment matrix is often observed to wrap around the organic groundmass because it is deposited before lithification and hence compaction pushes the sediments around the *diagenetic solid bitumen* parallel to the bedding fabric of the rock^30,31^ (Fig. 2a).

Hackley et al.^11^, have defined an arbitrary reflectance limit of 0.30% in mechanically polished samples to differentiate *bituminite* (< 0.30%) from solid bitumen. However, Synnott et al*.*^30,31^ have shown that *bituminite* could be subject of intense biodegradation that leads to increase in Ro of up to 0.8%. This is further supported by the findings of Stasiuk and Goodarzi^43^ who determined that bituminite macerals can have reflectances in excess of 0.8%Ro. Therefore, a biodegraded *bituminite* is often mistaken as a solid bitumen in immature samples^11^, leading to further confusions over the genesis of these organic matter.

*Diagenetic solid bitumen* and its precursor, *bituminite/amorphinite* are suggested to be part of the oil-generative fraction of the kerogen, which following the increased thermal maturity, could potentially generate bitumen and oil^11^ (Fig. 1). However, the hydrocarbon generation potential of this OM depends mainly on the degree of bacterial alteration^31^. Continued increase of thermal stress during the catagenesis stage, may increase the reflectance of *diagenetic solid bitumen* along with other types of later phase solid bitumens. While *diagenetic solid bitumen* is originally formed at Ro < 0.5% (< 0.3%)^11^, it may no longer be distinguished in the samples from other forms of catagenetically formed solid bitumens at higher thermal maturity.

Since *diagenetic solid bitumen* is formed in the low temperature, it typically does not form a vacuolated texture, which is commonly observed due to rapid devolatilization. Instead, *diagenetic solid bitumen* appears microscopically as a relatively condensed, bulky amorphous mass^30,31^. However, depending on its hydrocarbon generation potential, continued thermal cracking in the catagenesis stage may result in evasion of gas and the development of vacuolated structure.

### Initial-oil solid bitumen

In this paper, *initial-oil solid bitumen* is a proposed nomenclature for the first catagenically formed solid bitumen evolved at the incipient (initial) oil generation window (Ro ~ 0.5–0.7%)^12,15^ (Table 1, Fig. 1). *Initial-oil solid-bitumen* is attributed to the Curalie’s^7^ “pre-oil solid bitumen” because it evolves, prior to the onset of the liquid crude oil (Table 1; Fig. 1).

At the early catagenesis stage (Ro ~ 0.5–0.7%), primary cracking of oil-prone organic matter leads to exudation of heavy, viscous bitumen, which is a precursor of crude oil. This early generated bitumen has limited ability to migrate and may only disseminate into proximal pores and cavities^15^. Organic petrologists have long recognized this early-generated bitumen as the exsudatinite maceral, which is a brownish (in white reflected light microscopy), often yellow fluorescing, viscous, amorphous matter^41,44^. This early bitumen, although formed before generation of the liquid crude oil, in fact is generated catagenically within the incipient oil generation window^7,8^. Further consolidation of this bitumen leads to formation of solid, amorphous, grey color (in white, reflected light microscopy), often non-fluorescing, organic matter, which here is referred to as the *initial-oil solid-bitumen*^11,45,46^ (Table 1; Fig. 1).

*Initial-oil solid-bitumen* is in essence the same matter that has been largely referred to as the “pre-oil solid bitumen” in the existing genetic classification^7,8^. However, the “pre-oil” contradicts the fact that this solid bitumen is actually generated within the oil window and further obscures the distinction with the *diagenetic solid bitumen*, which is evolved before the oil window (Table 1; Fig. 1).

Since *initial-oil solid-bitumen* is the product of the bitumen that is generated from primary thermal cracking of kerogen, it is associated with a substantial volume increase within the confined mineral matrix of a rock^15^. The thermal expansion of generated bitumen results in impregnation of the groundmass with bitumen and development of submicron parting separations parallel to the bedding fabric of the rock^15^. The generated bitumen and resulting solid bitumen is often spatially associated with its precursor oil prone organic matter and can occupy the place of its kerogen precursor with no transport involved (Fig. 2b). I*nitial-oil solid-bitumen* could also show microscopic evidence of flow but lacks a pervasive dissemination throughout the samples or any long-distance, regional migrations^8,46^ (Fig. 2b).

### Primary-oil solid bitumen

*Primary-oil solid bitumen* is a proposed name for the most prominent phase of solid bitumen formed during the primary-oil generation window (Ro ~ 0.7–1.0%) (Table 1; Fig. 1). The continued thermal degradation of fluid or solid OM, induce precipitation of heavy asphalt compounds^18,20,21,47–49^.

Two mechanisms have been suggested to form this phase of solid bitumen. In the first process, continued thermal stress in the primary-oil window results in generation of an extensive network of bitumen in the organic-rich source rocks^15,17^. The amount of oil-prone organic matter in the source rock is a critical aspect for the bitumen network to be developed. Lewan^15^ suggested that development of bitumen network in the primary-oil generation window is a prerequisite to the generation of liquid oil. Solidification of this bitumen network due to further thermal alteration, results in formation of the *primary-oil solid bitumen*, which resembles an extensive pervasive distribution in organic-rich source rocks^45,50^ (Fig. 2c, d).

In the second case, *primary-oil solid bitumen* is derived from the early maturity crude oil that is expelled and possibly migrated further into the tight and/or conventional reservoir rocks (e.g., Lower Triassic Montney tight gas reservoir^13,18,19,51^). Crude oil is a colloidal cocktail that can readily dissociate into different fractions (including asphalt) and that these fractions then mature differently with increasing thermal stress. Asphalt precipitation is induced primarily by either changing PVT conditions or more likely by the natural introduction of gas enriched in light n-alkanes (natural deasphalting). The precipitated asphalt may solidify as temperature drops when sample is brought to the surface^3^. Alternatively, precipitated asphalt could be solidified in the reservoir temperature as the result of continued thermal devolatilization^22,27,28,33–36^ (Table 1; Fig. 1).

**Figure 3:**
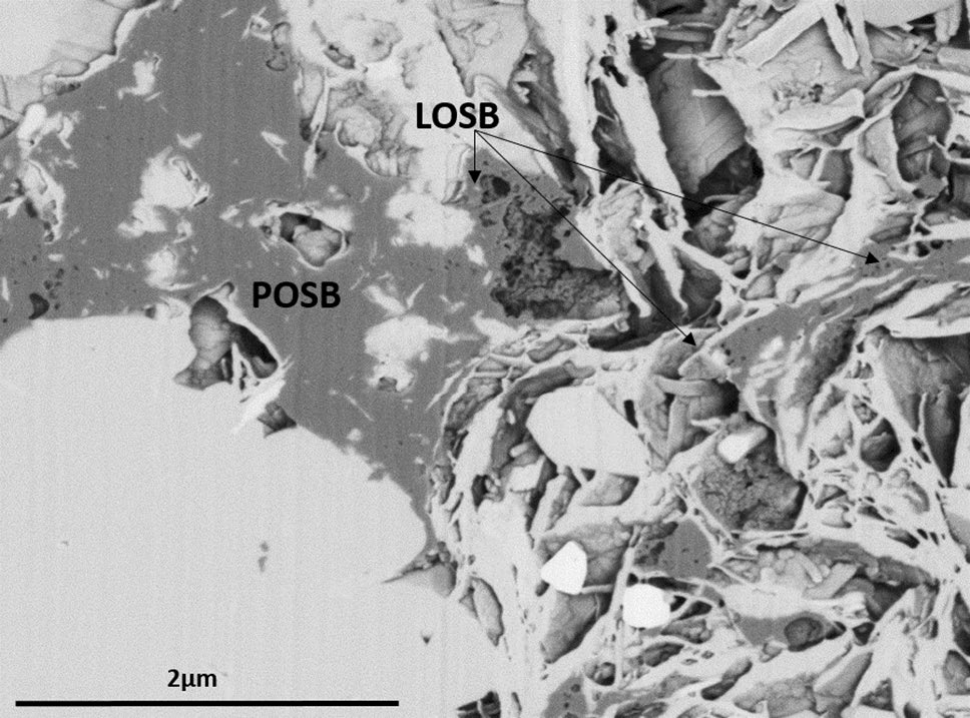
Focused Ion Beam-Scanning Electron Microscope (FIB-SEM) of the Lower Cretaceous Montney tight siltstone showing distinct spatial distributions of highly condensed *primary-oil solid bitumen* (POSB) in the largest paleo-pores and the highly vacuolated, less condensed, *late-oil solid bitumen* (LOSB) in the smaller pores (modified after Wood et al.^19^).

**Figure 4:**
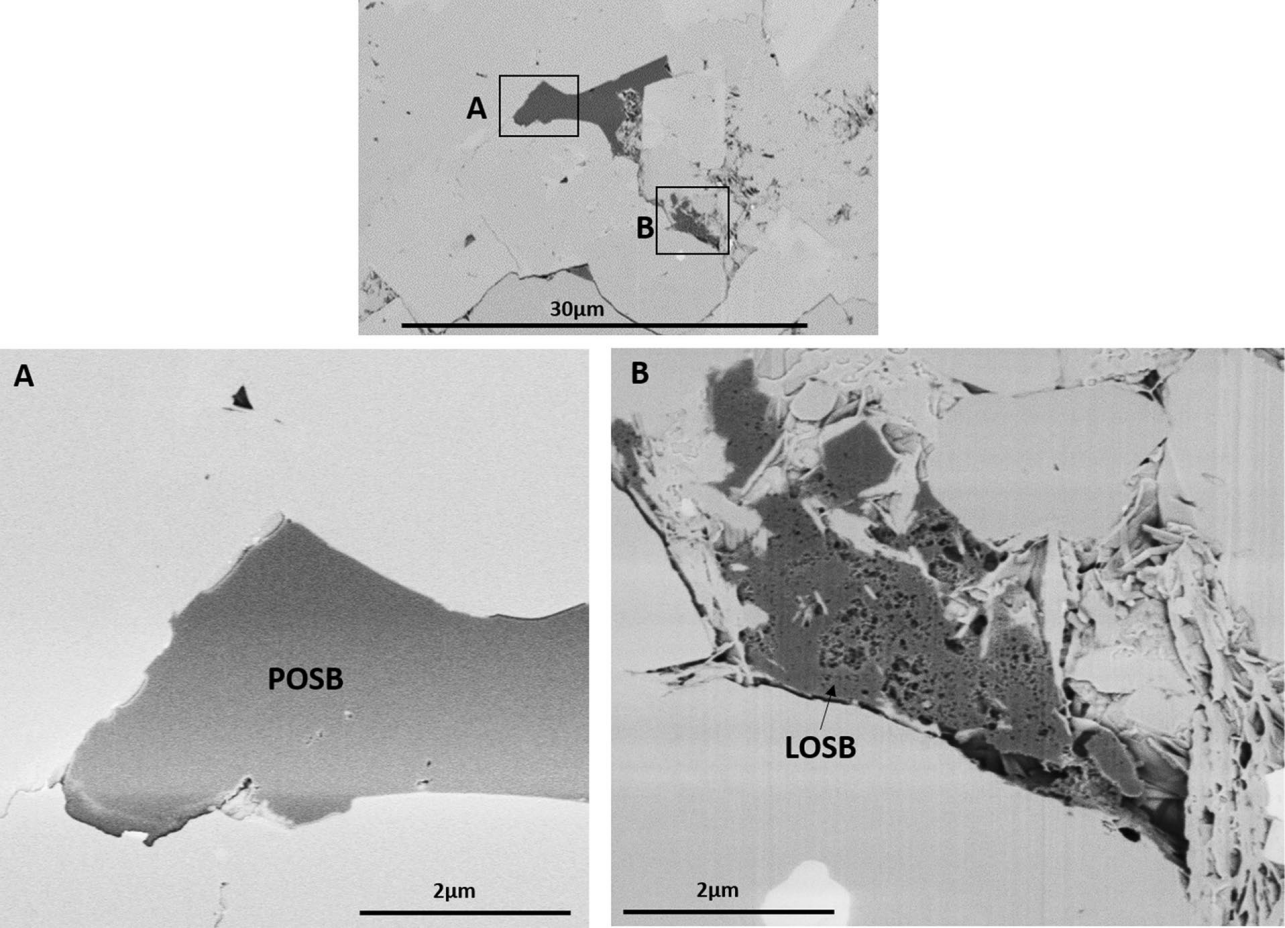
Focused Ion Beam-Scanning Electron Microscope (FIB-SEM) of the Lower Cretaceous Montney tight siltstone showing coexistence of condensed *primary-oil solid bitumen* (POSB) with the *late-oil solid bitumen* (LOSB), which has appeared to penetrate into the tighter pores (modified after Sanei et al.^55^).

Examples of the *primary-oil solid bitumen* have been reported by Wood et al.^18,19^ showing: (1) the void-filling and globular/granular bitumen in the Montney tight reservoir formed by the early precipitation of asphalt floccules from an unstable bitumen or precursor oil phase in the central portions of large open pores. With further burial and increase in thermal stress, a globular/granular texture asphalt fraction matured and consolidated into solid bitumen (Figs. 3, 4). Similar globular/granular OM aggregates have also been reported in other source rocks^52,53^. (2) The fractures-filled solid bitumen are commonly observed in many tight and shale formations, which is formed by incremental viscous flow of oil/bitumen through the fractures. This results in flow structure and caused matrix grains and clays to spall from the groundmass and to become mobilized/entrained within the fractures. Fractures-filled solid bitumen reflect the widespread migration of bitumen and/or liquid petroleum through open fracture systems^9,18,19^ (Fig. 2d).

Physical and chemical properties of the precursor oil and bitumen defines migration range, distribution, and overall physical properties of the resulting solid bitumen found in source rocks and tight reservoirs. The migration range of solid bitumen is a direct response to the net increase in volume of the organic components within a confined mineral matrix due to thermal cracking of kerogen and expansion of the precursor bitumen and oil products. The volume of products generated by thermal cracking of petroleum is typically greater than the initial volume of the kerogen^54^. Lewan^15^ demonstrated that increase in volume of the organic phase was accommodated within the mineral matrix by generation of submicron parting separations of bitumen from its precursor organic matter at the incipient oil window. As thermal stress increases, a continuous bitumen network is developed, which is a precursor to generation of liquid petroleum and its expulsion into adjacent fractures and pore networks. Therefore, the resulting *primary-oil solid bitumen* is characterized by its pervasive nature, forming a network throughout the rock matrix (Fig. 2d). Lewan^15^ noted that the quantity of oil-prone organic matter within a rock directly controls the amount of volume expansion and hence migration of the generated bitumen. Increase in the quantity of oil-prone organic matter would lead to forming a network of bitumen and hence more pervasive distribution within the pore network of the rock.

### Late-oil solid bitumen (solid-wax or ozocerite)

As thermal maturity increases, the early maturity crude oil increasingly fractionates and the asphalt dissociates from the more hydrogen-rich, mature paraffin-rich heavy oil^20^ (Table 1; Fig. 1). This mature, high-wax oil has lower viscosity than the early maturity crude oil and hence is able to intrude into the tighter pore networks within the source/tight reservoir rocks. When this paraffin-rich oil migrates to a temperature below its cloud point, the wax will be then crystallized and precipitate^3^. The high wax oil that is liquid at a reservoir temperature (~ 90 °C), can be solidified to a solid-wax, ozocerite at the surface temperature^3^. Thermal or chemical reactions that lead to the oxidation/degradation/dehydrogentaton of the remaining waxes in source/tight reservoir rocks could also result in consolidation of wax. Bacterial sulfate reduction in uplifted basins and thermochemical sulfate reduction are examples of these non-thermal cracking processes that would lead to the formation of solid-wax from a precursor waxy bitumen^29^. This solid-wax or ozocerite designated in this study as *late-oil solid bitumen* (Table 1; Fig. 1).

Petrographically, *late-oil solid bitumen* is characterized as amorophous, intergranular, solid (soft to hard polishing hardness), grey (in white-reflected light microscopy), and non-fluorescing (in UV-reflected light microscopy). *Late-oil solid bitumen* exhibits pervasive dissemination in finer pore networks compared to its *primary-oil solid bitumen* counterpart. Sanei et al.^55^ reported two types of solid bitumens commonly found within intergranular pores in the Lower Triassic Montney tight reservoir (siltstone): (1) highly condensed solid bitumen mass accumulation with virtually no nanoporosity and higher reflectivity (BRo), distributed in the larger intergranular spaces (paleopores), and (2) highly vacuolated solid bitumen with extensive nanoporosity and lower reflectivity (BRo), distributed in the smaller paleopores. These two types of solid bitumen were commonly observed a few microns apart and connected to each other by a stringer of solid bitumen (Fig. 3, 4).

The first type of solid bitumen is related to the first generation phase of crude oil, which has the highest viscosity and its flow is slow and restricted by the size and quality of the pore network. Therefore the asphalt fraction that arose through dissociation of this crude oil tends to be distributed only in the largest paleo-pores in the host rock at the time of the migration^18,19,51^. This precipitated asphalt later consolidated to form the *primary-oil solid bitumen*, which is highly condensed and consolidated due to heavy molecular nature of its precursor asphalt (Figs. 3, 4).

The second type of solid bitumen is related to the later generation phase of crude oil. Increasing thermal maturity within the oil window leads to increase in hydrogen content and decrease in the molecular size and viscosity of the generated mature oil. As a result, *late-oil solid bitumen* tends to penetrate further into tighter pores that have not been already occupied by the previous phase solid bitumen. This results in distinct spatial distribution between the two phases of solid bitumen, largely controlled by the size and quality of the paleopore network at the time of migration (Figs. 3, 4).

The *late-oil solid bitumen*, tends to show lower mean reflectance as it is formed at later stage of thermal maturity and is physically less condensed since it is derived from the lower asphalt content, lower molecular size bitumen. This would cause distinct petrographic characteristics for the *late-oil solid bitumen* including: more halo, vacuolated structure, often lower reflectance, with preferential distribution in the finer interstices of the pore network. The *late-oil solid bitumen* can be distinguished from its more condensed, bulky, higher reflecting, earlier phase counterpart, *primary-oil solid bitumen*, which often shows preferential distribution in the larger pores (Figs. 3, 4).

### Pyrobitumen

*Pyrobitumen* is not a new product but rather the gas- and non-generative form of previously formed solid bitumen, produced due to intense thermal alterations in the dry gas window and higher (Ro > 1.4%^8^) (Table 1; Fig. 1). Both secondary thermal cracking and thermochemical sulfate reduction of the remaining hydrocarbon residue and solid bitumens in source/tight reservoir rocks can potentially lead to formation of *pyrobitumen*^8,29,56^ (Fig. 1).

Further thermal cracking of solid bitumen leads to generation of gas until depleting its entire potential gaseous hydrocarbon and evolves into a high reflecting, mainly non-generative, *pyrobitumen* (epi-impsonoite^20^). The Ro > 0.7% threshold for generation of epi-impsonite (*pyrobitumen*) by Jacob^20^, is too low and is likely attributed to the onset of the first phase of solid bitumen after generation of oil, which is identified here as *primary-oil solid bitumen*. *Pyrobitumen*, is considered a mainly non-generative form of solid bitumen and recent work has shown its evolution at much higher reflectance (Ro > 1.5%^8^). Therefore Jacob’s^20^ defined epi-impsonite is not the exact equivalent of *pyrobitumen*^8^.

*Pyrobitumen* has an important role in hydrocarbon storage as it is related to its porosity and pore size distribution. Substantial pore networks are interpreted to develop within the *pyrobitumen* as a result of thermal cracking and subsequent evasion of gas (devolatilization) from its precursor solid bitumen at increased levels of thermal maturity^8,57–59^. Mastalerz et al.^57^ demonstrated increase in porosity and pore size distribution as a result of maturation of solid bitumen in particular in the post-mature stages. At immature and early oil window, solid bitumen does not develop substantial amount of porosity^46^. When maturity reaches the gas window stage, a widespread network of porosity is developed within the pyrobitumen. These pores serve as major sites of gas storage in the shale and tight reservoirs^58^.

## Conclusions

This paper presents a new schematic model of generation and timing of bitumen and solid bitumen throughout the continuum process of thermal maturation in source and tight reservoir rocks.

Bitumen is a highly viscous, extractable, semi-fluid hydrocarbon residue (FHR) often seen throughout the entire diagenesis and catagenesis stages. Bitumen evolves through one of three ways: (1) during the diagenesis stage from a primary deposition of flocculent lipid organic matter (*bituminite/amorphinite/prebitumen*), (2) as a secondary product exuding from the oil prone organic matter in the incipient oil window, (3) as the natural deasphalting and wax precipitation products of crude oil throughout the primary and late oil windows.

Solid bitumen is a consolidated product of bitumen that has undergone further bacterial and/or thermal degradation. Solid bitumens are genetically classified into five distinct types: (1) *diagenetic solid bitumen (or degraded bituminite)*, which is derived from biodegradation or low temperature alterations of *bituminite* in the diagenesis stage (Ro < 0.5%); (2) *Initial-oil solid bitumen*, which is a consolidated product of bitumen exuded (exsudatinite) from early thermal cracking of organic matter at the incipient oil window (Ro ~ 0.5–0.7%); (3) *Primary-oil solid bitumen*, a pervasive form of solid bitumen derived from either the bitumen network generated after continued thermal cracking of kerogen in the organic rich source rocks, or the deasphalting of the early maturity crude oil in the tight reservoirs (Ro ~ 0.7–1%); (4) *Late-oil solid bitumen* (solid-wax or ozocerite) is formed primarily due to consolidation of waxy bitumen derived from wax crystallization of the mature paraffinic heavy oil at the primary- and late-oil generation windows (Ro ~ 0.8–1.4%); and (5) *Pyrobitumen*, which is a mainly non-generative solid bitumen evolved from thermal cracking of the remaining hydrocarbon residue of the aforementioned four types of solid bitumens in the dry gas window (Ro > 1.4%).

The nature of generated bitumen and oil defines the migration range, pore distribution, and overall physical properties of the resulting solid bitumens in source rocks and tight reservoirs.

This model shows continuous generation of oil and bitumen throughout the oil window, which explains the occurrence of multi phases of solid bitumen. Earlier phases of solid bitumen may coexist with later generated phases of solid bitumen, bitumen, and oil in a single sample at any time during the oil window. However, in the gas window, continued thermal stress leads to conversion of all forms of earlier formed FHR and solid bitumens into *pyrobitumen*.

## Data Availability

This manuscript is based on synthesis and analysis of the previously published data. Further information that support the findings of this study are available from the corresponding author upon reasonable request.
